# Sociodemographic Differences in Physician-Based Mental Health and Virtual Care Utilization and Uptake of Virtual Care Among Children and Adolescents During the COVID-19 Pandemic in Ontario, Canada: A Population-Based Study

**DOI:** 10.1177/07067437231156254

**Published:** 2023-02-28

**Authors:** Alene Toulany, Paul Kurdyak, Therese A. Stukel, Rachel Strauss, Longdi Fu, Jun Guan, Lisa Fiksenbaum, Eyal Cohen, Astrid Guttmann, Simone Vigod, Maria Chiu, Charlotte Moore Hepburn, Kimberly Moran, William Gardner, Mario Cappelli, Purnima Sundar, Natasha Saunders

**Affiliations:** 1 7979 The Hospital for Sick Children, Toronto, Canada; 2 Department of Pediatrics, 7938 University of Toronto, Toronto, Canada; 3 ICES, Toronto, Canada; 4 Child Health Evaluative Sciences, SickKids Research Institute, Toronto, Canada; 5 Institute of Health Policy, Management and Evaluation, 7938 University of Toronto, Toronto, Canada; 6 Department of Psychiatry, Temerty Faculty of Medicine, 7938 University of Toronto, Toronto, Canada; 7 Edwin S.H. Leong Centre for Healthy Children, 7938 University of Toronto, Toronto, Canada; 8 Centre for Addiction and Mental Health, Toronto, Canada; 9 Women's College Hospital and Women's College Research Institute, Toronto, Canada; 10 Children's Mental Health Ontario, Toronto, Canada; 11 Children's Hospital of Eastern Ontario Research Institute, Ottawa, Canada; 12 School of Epidemiology and Public Health, 6363 University of Ottawa, Ottawa, Canada; 13 460386 Knowledge Institute on Child and Youth Mental Health and Addictions, Ottawa, Canada

**Keywords:** mental health, virtual care, digital, telemedicine, pandemic, COVID-19, equity, immigrant

## Abstract

**Objective:**

We sought to evaluate the relationship between social determinants of health and physician-based mental healthcare utilization and virtual care use among children and adolescents in Ontario, Canada, during the COVID-19 pandemic.

**Methods:**

This population-based repeated cross-sectional study of children and adolescents (3–17 years; *N* = 2.5 million) used linked health and demographic administrative data in Ontario, Canada (2017–2021). Multivariable Poisson regressions with generalized estimating equations compared rates of outpatient physician-based mental healthcare use during the first year of the COVID-19 pandemic with expected rates based on pre-COVID patterns. Analyses were conducted by socioeconomic status (material deprivation quintiles of the Ontario Marginalization index), urban/rural region of residence, and immigration status.

**Results:**

Overall, pediatric physician-based mental healthcare visits were 5% lower than expected (rate ratio [RR] = 0.95, 95% confidence interval [CI], 0.92 to 0.98) among those living in the most deprived areas in the first year of the pandemic, compared with the least deprived with 4% higher than expected rates (RR = 1.04, 95% CI, 1.02 to 1.06). There were no differences in overall observed and expected visit rates by region of residence. Immigrants had 14% to 26% higher visit rates compared with expected from July 2020 to February 2021, whereas refugees had similarly observed and expected rates. Virtual care use was approximately 65% among refugees, compared with 70% for all strata.

**Conclusion:**

During the first year of the pandemic, pediatric physician-based mental healthcare utilization was higher among immigrants and lower than expected among those with lower socioeconomic status. Refugees had the lowest use of virtual care. Further work is needed to understand whether these differences reflect issues in access to care or the need to help inform ongoing pandemic recovery planning.

## Résumé

**Objectif:**

Nous cherchions à évaluer la relation entre les déterminants sociaux de la santé et l’utilisation des soins de santé mentale basée sur un médecin et le recours aux soins virtuels chez les enfants et les adolescents de l’Ontario, Canada, durant la pandémie de la COVID-19.

**Méthodes:**

Cette étude transversale répétée dans la population des enfants et des adolescents (3–17 ans; N = 2,5 millions) utilisait des données liées administratives de santé et démographiques en Ontario Canada (2017–2021). Des régressions multivariables de Poisson avec des équations d’estimation généralisée comparaient les taux de l’utilisation ambulatoire des soins de santé mentale basée sur un médecin durant la première année de la pandémie de la COVID-19, avec des taux prévus basés sur les modèles pré-COVID. Les analyses ont été menées par statut socio-économique (quintiles de privation matérielle de l'Indice de marginalisation de l'Ontario), par région de résidence urbaine/rurale, et par statut d’immigrant.

**Résultats:**

Globalement, les visites de soins de santé mentale basées sur un médecin pédiatre étaient 5 % moins nombreuses que prévu (RR = 0,95, IC à 95 % 0,92 à 0,.98) chez ceux qui habitaient les régions les plus démunies la première année de la pandémie, comparé aux moins démunis avec 4 % plus que les taux prévus (RR = 1,04, IC à 95 % 1,02 à 1,06). Il n’y avait pas de différences dans les taux généraux observés et prévus par région de résidence. Les immigrants avaient des taux de visites 14-26 % plus élevés comparés aux prévisions de juillet 2020 à février 2021, tandis que les réfugiés avaient des taux observés et prévus semblables. L’utilisation des soins virtuels était d’environ 65 % chez les réfugiés, comparé à 70 % pour toutes les couches.

**Conclusion:**

Durant la première année de la pandémie, l’utilisation des soins de santé mentale basée sur un médecin pédiatre était plus élevée chez les immigrants et plus faible que prévu chez ceux ayant un statut socio-économique plus faible. Les réfugiés avaient l’utilisation la plus faible des soins virtuels. Il faut plus de recherche pour comprendre si ces différences reflètent des problèmes d’accès aux soins ou s’il faut contribuer à éclairer la planification de la récupération de la pandémie.

## Introduction

School closures and other public health measures during the COVID-19 pandemic isolated many children and adolescents from their peers and significantly disrupted their routines.^1,2^ Concerns about an increase in mental health problems in this group have emerged due to a combination of risk factors, including loss of essential support, social isolation, lack of control, fear of infection, changes in access to care, and parent/caregiver stress.^3,4^ Despite this, there has been only a modest increase in physician-based pediatric outpatient mental healthcare use during the pandemic, accompanied by a rapid shift to predominantly virtual modes of care delivery.^5^

A growing body of research identifies social and structural determinants such as educational attainment, poverty, immigration/refugee status, and race and/or ethnicity at the root of inequities in mental healthcare, many of which have been amplified during the pandemic.^6–8^ It is important to recognize that it is the discrimination and structural inequities experienced by these marginalized groups that contribute to poor health outcomes, rather than demographic characteristics (e.g. race/ethnicity) themselves. Furthermore, children and adolescents from marginalized communities have likely experienced disproportionate stress from both the direct (e.g. exposure to infections and their sequelae) and indirect (e.g. economic hardship) effects of the pandemic on their families and social networks.^1,6^ Higher prevalence rates and more pronounced depressive symptoms have been observed among adult populations with lower incomes and greater stress levels during the pandemic.^9^ In addition, pediatric immigrants and refugees have greater difficulty accessing the mental healthcare system in Ontario due to stigma, cultural factors, and health literacy.^10,11^

While social determinants can impact the health of populations, they can also impact access to health services. Food and/or housing insecurity,^12,13^ precarious employment,^14^ inflexible schedule work or childcare schedules,^13,15^ and limited regional availability of healthcare resources contribute to some of the challenges faced by certain population groups in accessing quality healthcare. Furthermore, while the significant increase in virtual care during the pandemic^5^ may reduce barriers to access such as geography, difficulty getting time off school or work, or transportation costs, it may introduce others, including the need for reliable internet, a computer or other device to connect, and a private space.^16–19^ The shift to virtual care, therefore, risks increasing health disparities, particularly for marginalized groups including refugees who may not be able to afford or have access to high-speed internet in their area or have the digital literacy to navigate it.^20–23^

Pediatric mental healthcare in Ontario is characterized by high patient needs, significant service deficiencies, and the absence of a standardized system for measuring child and adolescent mental health.^24^ The majority of mental health diagnoses and prescription management for children and adolescents in Ontario is performed by physicians in Ontario,^25^ in contrast to mental health clinicians and therapists in the private sector or community children's mental health agencies. In this population-level study, we evaluated the relationship between a set of measurable social determinants of health (area-based measures of socioeconomic status, urban/rural region of residence, and immigration status) and utilization rates of physician-based mental healthcare and virtual care use among children and adolescents during the first year of the COVID-19 pandemic in Ontario, Canada, a publicly funded universal healthcare system. We hypothesized that outpatient physician-based mental healthcare and virtual care use would be less among those living in the most deprived neighbourhoods and among refugees.

## Materials and Methods

### Study Design and Population

This was a population-based repeated cross-sectional study of physician-based mental health visits considering all children and adolescents ages 3–17 years living in Ontario and eligible for provincial health insurance using linked data from ICES, an independent, nonprofit research institute whose legal status under Ontario's health information privacy law allows it to collect and analyze healthcare and demographic data, without consent, for health system evaluation and improvement. We identified all physician-based pediatric mental health-related visits before (January 1, 2017, to February 29, 2020) and during (March 1, 2020, to February 28, 2021) the COVID-19 pandemic. Non-Ontario residents, individuals with invalid birth dates and deaths within the study period, and those with missing data on sex were excluded from this study.

### Data Sources

We accessed health and demographic administrative data from several databases, which were linked using unique ICES-encoded identifiers. We used the Ontario Health Insurance Plan (OHIP) physician claims database for insured services for outpatient visits. The Registered Persons Database (RPDB) developed and maintained by the Ministry of Health, captured sociodemographic variables. The RPDB includes the date of birth, sex, and postal code of all Ontario residents eligible for OHIP. Statistics Canada's Postal Code Conversion File linked postal codes from RPDB to Canadian Census data.

### Outcome Measures and Exposure

Our primary outcome was the number of physician-based outpatient mental health-related visits (e.g. mental health concerns and diagnoses) to a family physician, pediatrician, or psychiatrist. We modelled expected visit rates during the pandemic period based on 3-year prepandemic trends and compared observed to expected monthly visit rates during the pandemic period. Mental health-related visits were categorized as either in-person or virtual (i.e. telephone or video) based on validated mental health physician service billing codes^26^ (eTable 1 in the online supplemental material) widely used for mental health system performance reporting in Ontario and Canada.^27^ In response to the government's efforts to limit the spread of COVID-19 in Ontario, widespread virtual visit billing codes (telephone and video) came into effect in Ontario on March 14, 2020.^28^ Diagnostic codes used in physician billings during the visit were based on the *International Classification of Diseases, 8th Revision*. Visit rates were expressed per 1,000 population overall and by three social determinants of health: neighbourhood socioeconomic status as measured by material deprivation, urban/rural region of residence, and individual immigration and refugee status. Material deprivation, a validated geographically (census) based component of the Ontario Marginalization index, incorporates indicators of income, quality of housing, educational attainment, and family structure from the 2016 Canadian Census and is reported in quintiles, with the lowest quintile representing geographic areas that are least deprived and the highest quintile representing the most deprived.^29,30^ Geographic areas are based on the Census dissemination areas, the smallest geographic unit of 400 to 700 residents for which Canadian Census data are available. Urban/rural region of residence was based on the Rurality Index of Ontario for the RPDB data, calculated on 3 weighted components: population density, travel time to the nearest advanced referral centre, and travel time to a basic referral centre and dichotomized as rural or urban.^31^ Immigration status (immigrants, refugees, and nonimmigrants) was based on the Immigration, Refugees and Citizenship (IRCC) Permanent Resident Database, which includes landing records for permanent legal immigrants to Ontario from January 1995 to May 2017. Immigrants with permanent resident status are eligible for provincial health insurance after living in Ontario for 3 months, asylum seekers receive federal health insurance benefits and those who are successful in their refugee claims are eligible after a 3-month waiting period, while refugees who arrive in Ontario with permanent resident status (i.e. resettled refugees) are immediately eligible.^32^ Based on 2016 Canadian Census data, 21.9% of the population were foreign-born (i.e. immigrants), and among Canadian jurisdictions, Ontario has the largest proportion. Immigrants are selected based on 3 admission categories: economic (i.e. to fill labour market needs), sponsored by family (i.e. family reunification), and refugees (i.e. humanitarian or compassionate needs).^33^ Approximately 60% of immigrants are admitted under the economic category, and many are racialized.^8^ About 52% of nonrefugee immigrants in Ontario come from South and East Asia, whereas most refugees come from South Asia and Africa and a majority of all recent newcomers would be considered racialized in Canada.^10,11^ Linkage of IRCC data to population health registries has a linkage rate of 86% in Ontario.^34^ The proportion of visits involving virtual care was assessed as a secondary outcome.

The exposure was the first 12 months of the COVID-19 pandemic, defined as March 1, 2020, to February 28, 2021, representing the onset of the pandemic in Ontario to the end of complete data availability. Virtual and in-person physician-based mental healthcare utilization was compared before and during the COVID-19 pandemic, and the uptake of virtual mental healthcare by sociodemographic characteristics was examined.

### Statistical Analyses

Characteristics of the study population are described using means (standard deviation [SD]), frequencies, and percentages. To determine the proportion of virtual mental health visits during the pandemic, we calculated the number of overall weekly outpatient visits with a virtual care fee code relative to the total weekly outpatient visits. To measure differences in mental healthcare use across social determinants of health strata, we used Poisson generalized estimating equations models for clustered count data to model 3-year pre-COVID trends and used these to forecast expected post-COVID trends in the absence of restrictions, separately for each stratum, as in previous work.^5^ The unit of analysis was the age group-sex-week stratum. The dependent variable was the stratum-specific count of events to the population in the stratum; the offset was the log of the stratum-specific population; the working correlation structure was *AR*(1) autocorrelation with a lag of 1, to account for correlations in visit rates over time. The pre-COVID model included age group-sex indicators, a continuous linear term of weeks since January 1, 2017, to estimate the secular trend in visit rates through March 1, 2020, and pre-COVID month indicators to model monthly variations, with April as the reference month of the fiscal year.

Separately for each stratum, we computed the expected post-COVID visit rates (and 95% confidence intervals [CIs]) by applying the linear combination of pre-COVID regression coefficients to the post-COVID age-sex-month strata and exponentiating. The relative change in post-COVID visit rates was expressed as the ratio of observed to expected rates; these were obtained by calculating the difference between observed and expected post-COVID log rates, computing standard errors and 95% CIs for this difference as it was a linear combination of regression coefficients, and finally exponentiating to express rates and CIs on the original scale. For each stratum, we excluded observations with missing variable values from the analysis. Secondary analyses included stratifications of the main outcome measure by clinically relevant diagnostic groupings (eTable 1 in the online supplemental material): mood and anxiety disorders, psychotic disorders, substance use disorders, social problems, and neurodevelopmental and other disorders and stratifications by physician type: family physician/general practitioner, pediatrician, and psychiatrist.

All statistical analyses were done using SAS version 9.4 using procedure PROC GENMOD (SAS Institute, Cary, NC, USA). The use of these data was authorized under section 45 of Ontario's Personal Health Information Protection Act, which does not require review by a Research Ethics Board. Cell sizes <6 were suppressed to meet institutional policy. This study followed the Reporting of Studies Conducted Using Observational Routinely-Collected Data (RECORD) reporting guideline.^35^

## Results

Demographic characteristics of our study population were stable over the 3-year pre-COVID baseline period (Table 1). In 2021, there were 2,427,486 children and adolescents ages 3 to 17 years living in Ontario. Almost half (48.7%) were female (*n* = 1,181,864), and the mean age was 10.1 years (SD = 4.3). Ninety percent lived in urban settings (*n* = 2,185,925). Neighbourhoods with high material deprivation (quintiles 4 and 5) accounted for 35.0% of the population (*n* = 849,115). Nonimmigrants accounted for 89.8% (*n* = 2,180,258) of our study cohort, 3.4% (*n* = 83,717) were nonrefugee immigrants, and 1.1% (*n* = 26,346) came to Ontario as refugees.

**Table 1.** Baseline Demographic Characteristics of Children and Adolescents, Ages 3 to 17 Years in Ontario, 2017–2021.

Year	2017	2018	2019	2020	2021
Children and adolescents on January 1^st^, *N*	2,378,238	2,394,939	2,417,431	2,444,494	2,427,486
Age					
Mean ± SD	10.08 ± 4.31	10.08 ± 4.30	10.09 ± 4.29	10.10 ± 4.29	10.11 ± 4.28
Median (IQR)	10 (6–14)	10 (6–14)	10 (6–14)	10 (6–14)	10 (6–14)
Age group, *n* (%)					
3 to 12 years	1,572,454 (66.1)	1,585,340 (66.2)	1,599,458 (66.2)	1,616,460 (66.1)	1,600,708 (65.9)
13 to 17 years	805,395 (33.9)	809,212 (33.8)	817,672 (33.8)	828,034 (33.9)	826,383 (34.0)
Sex, *n* (%)					
Female	1,157,710 (48.7)	1,166,048 (48.7)	1,177,303 (48.7)	1,190,209 (48.7)	1,181,864 (48.7)
Male	1,220,528 (51.3)	1,228,891 (51.3)	1,240,128 (51.3)	1,254,285 (51.3)	1,245,622 (51.3)
Region of residence, *n* (%)					
Rural	232,896 (9.8)	233,949 (9.8)	235,736 (9.8)	238,331 (9.7)	237,012 (9.8)
Urban	2,139,999 (90.0)	2,155,898 (90.0)	2,176,616 (90.0)	2,201,245 (90.0)	2,185,925 (90.0)
Missing	5,343 (0.2)	5,092 (0.2)	5,079 (0.2)	4,918 (0.2)	4,549 (0.2)
Material deprivation quintile, *n* (%)					
1 (least deprived)	554,872 (23.3)	566,415 (23.7)	579,339 (24.0)	590,590 (24.2)	586,375 (24.2)
2	511,006 (21.5)	514,881 (21.5)	520,740 (21.5)	526,805 (21.6)	522,334 (21.5)
3	437,458 (18.4)	438,988 (18.3)	441,357 (18.3)	445,255 (18.2)	442,706 (18.2)
4	398,995 (16.8)	399,495 (16.7)	401,227 (16.6)	404,106 (16.5)	401,698 (16.5)
5 (most deprived)	448,842 (18.9)	448,083 (18.7)	447,352 (18.5)	450,297 (18.4)	447,417 (18.4)
Missing	27,065 (1.1)	27,077 (1.1)	27,416 (1.1)	27,441 (1.1)	26,956 (1.1)
Immigration status, *n* (%)					
Nonimmigrant/long-term resident	2,195,387 (92.3)	2,204,012 (92.0)	2,204,083 (91.2)	2,200,379 (90.0)	2,180,258 (89.8)
Refugee	40,339 (1.7)	37,276 (1.6)	33,586 (1.4)	29,987 (1.2)	26,346 (1.1)
Immigrant	142,510 (6.0)	128,072 (5.3)	112,348 (4.6)	98,237 (4.0)	83,717 (3.4)
Missing	27,065 (1.1)	27,077 (1.1)	27,416 (1.1)	27,441 (1.1)	26,956 (1.1)

*Note*. SD=standard deviation; IQR=interquartile range.

### Material Deprivation

**Figure 1.**
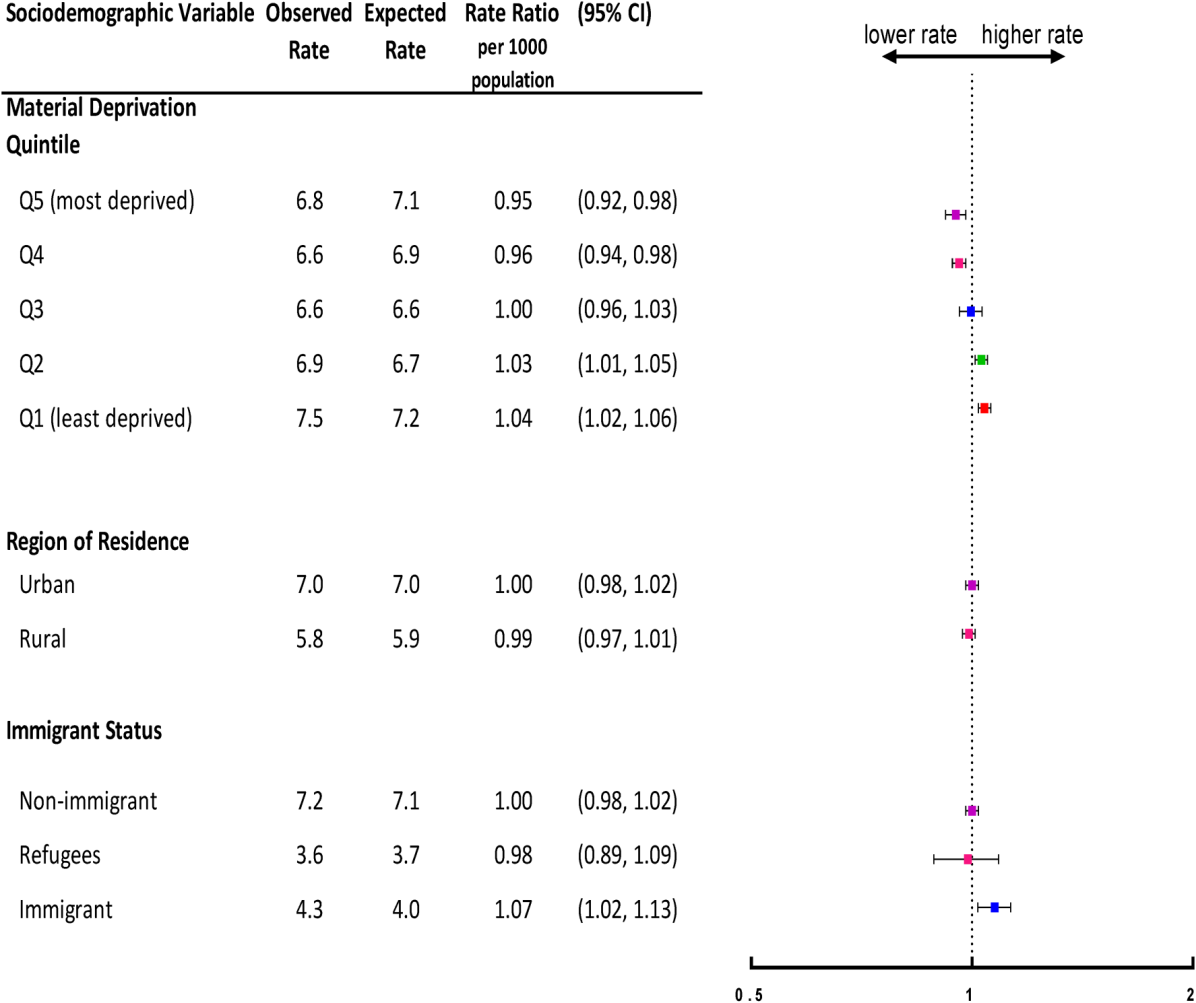
Adjusted overall rate ratio of observed mental health-related visit rates following the onset of the COVID-19 pandemic compared to the expected rates based on 3-year prepandemic rates, by neighbourhood-level material deprivation quintile, region of residence, and immigrant status in Ontario.

Figure 1 shows the gradient in observed versus expected overall visit rates during the first year of the pandemic by each sociodemographic variable. Overall, those living in the most deprived neighbourhood (i.e. quintile 5) had the lowest mental health-related visit rates (6.8 per 1,000 population) compared to expected (7.1 per 1,000 population; rate ratio [RR] = 0.95, 95% CI, 0.92 to 0.98). In contrast, those in the least deprived neighbourhood (i.e. quintile 1) had the highest visit rates (7.5 per 1,000 population) compared to expected (7.2 per 1,000 population; RR = 1.04, 95% CI, 1.02 to 1.06).

**Figure 2.**
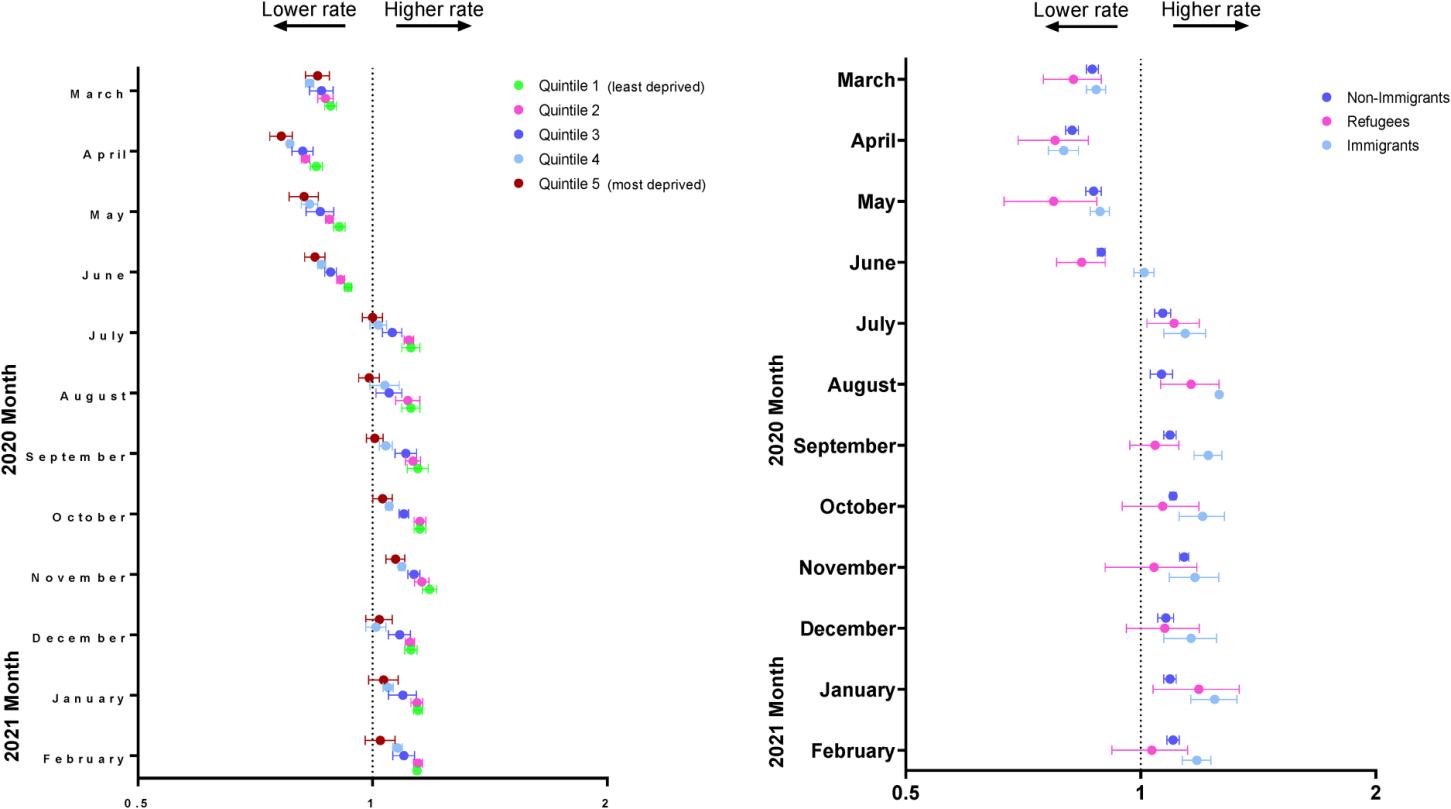
Adjusted monthly rate ratio of observed mental health-related visit rates following the onset of the COVID-19 pandemic compared to the expected rates based on 3-year prepandemic rates, by (a) material deprivation and (b) immigration status in Ontario.

Monthly rate ratios of observed mental health-related visit rates following the onset of the pandemic compared to the expected rates based on 3-year prepandemic rates are presented in Figure 2 and eTable 2 in the online supplemental material. For the first 3 months following the onset of the pandemic, across all material deprivation quintiles, observed rates of mental health-related visits were lower than expected; however, by July 2020, rates returned to expected or above expected levels for all quintiles. Also, in July 2020, observed rates of mental health-related visits were higher (6.5 per 1,000 population) than expected (5.8 per 1,000 population) among children and adolescents living in the least deprived neighbourhoods (RR = 1.12, 95% CI, 1.09 to 1.15). Conversely, children and adolescents living in the most deprived neighbourhoods (i.e. quintile 5) had equivalent observed (6.0 per 1,000 population) and expected visit rates in July 2020 (6.0 per 1,000 population; RR = 1.00, 95% CI, 0.97 to 1.03). Similar trends continued from August 2020 to February 2021.

**Figure 3.**
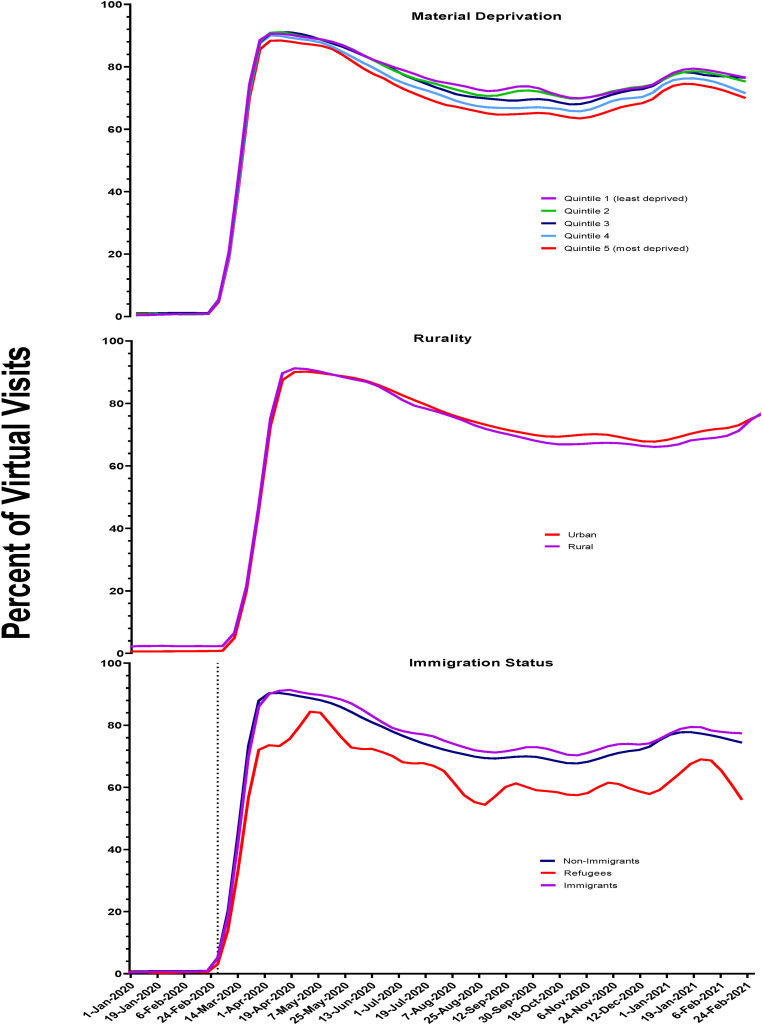
Proportion of virtual outpatient mental health visits by material deprivation, region of residence, and immigration status in Ontario.

There were slight differences in virtual care uptake by material deprivation quintiles (Figure 3). Overall, during the first year of the pandemic, the proportion of visits done virtually was lowest among those living in the most deprived neighbourhoods (quintile 5 = 72%) compared to the least deprived (quintile 1 = 77%).

### Region of Residence

Overall, in the first year of the pandemic, children and adolescents living in urban areas had the same observed mental health visits rates (7.0 per 1,000 population) compared to expected (7.0 per 1,000 population; RR = 1.00, 95% CI, 0.98 to 1.02) (Figure 1). Similarly, those living in rural areas had comparably observed (5.8 per 1,000 population) and expected (5.9 per 1,000 population mental health visit rates (RR = 0.99, 95% CI, 0.97 to 1.01) (eTable 4 in the online supplemental material).

In the 4 months following the onset of the pandemic, for both urban and rural residents, observed rates of mental health-related visits were less than expected. However, by July 2020, observed rates of mental health-related visits were higher (6.2 per 1,000 population) than expected (5.8 per 1,000 population) among children and adolescents living in the urban neighbourhoods (RR 1.07, 95% CI, 1.05 to 1.10) (eTable 4 in the online supplemental material). This trend continued until February 2021. For children and adolescents living in rural areas, observed mental health visits rates were higher (5.1 per 1,000 population) than expected (5.0 per 1,000 population starting in August 2020, and remained higher than expected until February 2021 (RR = 1.03, 95% CI, 1.02 to 1.04).

For both urban and rural residents, the proportion of virtual visits was similar throughout the first year of the pandemic at approximately 71% (Figure 3).

### Immigrant Status

Overall, in the first year of the pandemic, nonimmigrants had the highest and similar observed (7.2 per 1,000 population) and expected (7.1 per 1,000 population) mental health-related visits rates (RR = 1.00, 95% CI, 0.98 to 1.02). Overall, immigrants used physician-based outpatient mental health services more (4.3 visits per 1,000 population) than expected (4.0 visits per 1,000 population; RR = 1.07, 95% CI, 1.02 to 1.13). Refugees had the lowest observed and comparable expected mental health-related visit rates overall (3.6 and 3.7 per 1,000 population, respectively; RR = 0.98, 95% CI, 0.89 to 1.09) (Figure 1).

Monthly rate ratios of observed compared to expected of physician-based mental health visit rates by immigrant status are shown in Figure 2 and eTable 3 in the online supplemental material. Nonimmigrants had lower observed compared to expected visits rates from March to June 2020 and from July 2020 to February 2021 had higher observed compared to expected visit rates, peaking in November (9.2 observed vs. 8.1 expected per 1,000 population; RR 1.14, 95% CI, 1.12 to 1.15). Immigrants consistently had higher observed visit rates compared to expected from July 2020 to February 2021, peaking in August 2020 (RR = 1.26, 95% CI, 1.25 to 1.27). Similar to nonimmigrants and immigrants, refugees had lower observed than expected visit rates from March to June 2020. Following this, refugees had similarly observed and expected visit rates, except in July (RR 1.10, 95% CI, 1.02 to 1.19), August (RR 1.16, 95% CI, 1.06 to 1.126) and January (RR 1.18, 95% CI, 1.04 to 1.34).

Following the pandemic onset, virtual care accounted for approximately 76% of all visits for immigrants and nonimmigrants, whereas use by refugees was lower at 65% (Figure 3).

## Discussion

Consistent with our hypothesis, we found significant differences in observed rates of physician-based mental healthcare utilization compared to expected rates by a set of measurable social determinants of health during the first year of the COVID-19 pandemic among children and adolescents in Ontario. We found a modest gradient in physician-based outpatient mental healthcare utilization by material deprivation quintile and immigrant status compared to expected prepandemic rates suggesting that the health system responded, at least to some degree, to match the presumed increased need for services. In particular, nonrefugee immigrants had the highest overall rate of mental healthcare visits following the pandemic. Overall, children and adolescents living in the most deprived neighbourhoods had visit rates 5% lower than expected during the first year of the pandemic. Monthly observed visit rates were approximately 7% to 24% less than expected in the first quarter following the onset of the pandemic among all children and adolescents. In July 2020, observed visit rates were approximately 7% to 14% higher than expected for those living in the least deprived neighbourhoods, urban residents, immigrants, and nonimmigrants. This trend continued for the remainder of the study period, except for refugees who had similarly observed and expected visit rates for the majority of the time. With the rapid shift to virtual care, we found lower uptake among refugees and those living in the most deprived neighbourhoods. No differences were observed in the proportion of virtual mental healthcare visits by region of residence.

Our study casts light on overall disparities in the use of mental healthcare services and uptake of virtual care during the pandemic in Ontario. While the relative changes in rates compared to expected are modest, the impact across the population of ∼2.5 million children and adolescents living in Ontario is clinically significant as it poses further stress on a mental health system already at or over capacity. This has important implications for future mental health services, resources, funding, and capacity planning. Our data suggest that some marginalized groups may have experienced differential access to mental health services during the pandemic or had differing mental health needs. Although these sociodemographic differences and potential unmet needs likely existed prepandemic, they may have been exacerbated as a result of the pandemic. Our findings differ from Stephenson et al.,^36^ who found an equitable distribution of visits across various sociodemographic measures to family physicians before and during the pandemic in Ontario. In contrast, studies conducted in Canada before the pandemic reported barriers to accessing care for the children of immigrants^37^ and those living in areas with less physician supply.^38^ Importantly, before the pandemic, it has been suggested that there is a disparity such that more disadvantaged populations experience increased mental healthcare needs.^39,40^ This need is not necessarily met with increased utilization of mental health services;^39–41^ the reasons for this are unclear and may be due in part to differing baseline activities, supports, opportunities, and the ability to take time off work among disadvantaged families. During the pandemic, the impact of the loss may have been more profound for high-income families. These families may also have been better positioned to advocate for mental health services, have more flexible work schedules to attend appointments from home, and have the necessary digital literacy to navigate virtual care. Furthermore, we are unable to ascertain whether immigrants in our study had higher mental healthcare needs following the onset of the pandemic or were simply better able to access care. With approximately 60% of immigrants in Ontario being admitted under the economic category, many come with expert skills and education to fill labour market needs and may be well equipped to navigate the healthcare system.^8^

Before the pandemic, traditional telemental health models in Ontario were mostly limited to physicians enrolled with provincial networks servicing patients in remote locales. These services were rarely used, even among children and adolescents with high mental health needs.^18^ Measures taken to slow virus spread early in the pandemic required health systems globally to rapidly convert to virtual care. Early studies suggest that virtual modes of care delivery are a viable way to provide mental health services during the pandemic; however, equity, accessibility, and appropriateness need to be considered to ensure services are effective and meet the needs of children, youth, and their families.^42^

The results of our study are consistent with the existing literature that reports disparities in access to virtual care among refugees^22,23^ and those who are socially disadvantaged.^43–46^ To ensure equity in access, healthcare providers should deliver care in ways that match patient needs, recognizing that virtual care may introduce significant barriers. Intervention-generated inequalities are described within the public health literature as situations where healthcare interventions, designed to benefit the population, deliver greater benefits to advantaged populations.^47,48^ Virtual care is vulnerable to this phenomenon as many of the barriers introduced (e.g. access to stable internet and appropriate devices, digital literacy, and adequate space for private conversations) may disproportionately affect disadvantaged populations.^21^ Further qualitative research is needed to understand better the views and preferences of refugee and immigrant populations to help disparities in virtual mental healthcare uptake.

### Limitations

While our study's strength is the population-based nature of the health administrative data, these pose several limitations. Our study assesses physician-based mental healthcare utilization; however, we are unable to measure the population's need for mental healthcare or the quality of the healthcare services delivered. Lags in data transfer, coding accuracy, and lack of clinical detail are standard limitations of health administrative data. The paucity of individual-level socioeconomic variables in this study limits the extent to which inferences can be drawn about individual characteristics (e.g. living in a geographic region that is economically deprived is different from being economically deprived). Another major limitation of our study is that health administrative data does not measure all visits to nurses, or other allied health professionals including psychologists and social workers, who provide substantial community mental support. Our findings, therefore, only reflect physician-based services and may not generalize to the broader Ontario outpatient mental health system thereby potentially underreporting the full use of mental health services across the province. However, a substantial amount of pediatric mental healthcare in Ontario is delivered by physicians (approximately 3.9–6.4 visits per 100 population for adolescents between March 2020 and September 2021) as evidenced by our team's regular reporting on the province's mental health and addictions system performance measures.^25^ Further, while nonphysician-based care is not captured within our administrative datasets, only a portion of nonphysician-based care is publicly funded, with the remainder comprised of private practitioners who are accessible based on an ability to pay. This also speaks to a major limitation of our healthcare system in Ontario as there is a parallel private mental health system that cares for a small subset of the population. The out-of-pocket costs accrued by patients for nonphysician services are, unfortunately, substantial and groups with greater socioeconomic deprivation are less likely to have adequate private insurance that can help to mitigate these costs. Despite this, given the high prevalence of child and adolescent mental health concerns, strategies are needed to develop a robust standardized system for measuring pediatric mental health across the continuum of care and sectors to track outcomes and enable improved service planning at a health system level.

Moreover, virtual care fee codes precluded us from knowing what type of platform (e.g. telephone or video) was used by physicians. Finally, our results may not generalize to other health jurisdictions where remuneration may differ and affect the extent of virtual care uptake. Given that our data measures physician-based mental healthcare utilization through February 2021, future studies should examine if virtual care uptake continued throughout the pandemic.

## Conclusion

In the first year of the pandemic, physician-based pediatric mental healthcare utilization changes were observed across various measures of socioeconomic status in Ontario. Children and adolescents living in the most deprived areas used mental healthcare services less than expected, whereas those in the least deprived areas used services more than expected. Although no differences were observed by region of residence, immigrants had higher visit rates compared to expected during the pandemic, but refugees did not. The shift to virtual care also resulted in disproportionately limited access to care among refugees and those living in areas with high material deprivation. Our study highlights a potential imbalance between mental health needs and the use of services among children during the pandemic. System-level strategies are needed to mitigate barriers and disparities in mental healthcare access and uptake in virtual care during and following the COVID-19 pandemic for marginalized populations. Future work is needed to determine whether these inequities extend to populations beyond those with mental health concerns.

## Supplemental Material

sj-docx-1-cpa-10.1177_07067437231156254 - Supplemental material for Sociodemographic Differences in Physician-Based Mental Health and Virtual Care Utilization and Uptake of Virtual Care Among Children and Adolescents During the COVID-19 Pandemic in Ontario, Canada: A Population-Based Study Supplemental material, sj-docx-1-cpa-10.1177_07067437231156254 for Sociodemographic Differences in Physician-Based Mental Health and Virtual Care Utilization and Uptake of Virtual Care Among Children and Adolescents During the COVID-19 Pandemic in Ontario, Canada: A Population-Based Study by Alene Toulany, Paul Kurdyak, Therese A. Stukel, Rachel Strauss, Longdi Fu, Jun Guan, Lisa Fiksenbaum, Eyal Cohen, Astrid Guttmann, Simone Vigod, Maria Chiu, Charlotte Moore Hepburn, Kimberly Moran, William Gardner, Mario Cappelli, Purnima Sundar and Natasha Saunders in The Canadian Journal of Psychiatry
