# Supplementary material for: Sociodemographic Differences in Physician-Based Mental Health and Virtual Care Utilization and Uptake of Virtual Care Among Children and Adolescents During the COVID-19 Pandemic in Ontario, Canada: A Population-Based Study
Source: Can J Psychiatry. 2023 Feb 28;68(12):904–15. doi: 10.1177/07067437231156254 (PMC9982398; doi:10.1177/07067437231156254)
Supplement: sj-docx-1-cpa-10.1177_07067437231156254 - Supplemental material for Sociodemographic Differences in Physician-Based Mental Health and Virtual Care Utilization and Uptake of Virtual Care Among Children and Adolescents During the COVID-19 Pandemic in Ontario, Canada: A Population-Based Study [file sj-docx-1-cpa-10.1177_07067437231156254.docx]

**Supplementary Tables**

**eTable 1.** Ontario Health Insurance Plan and International Classification of Diseases, 8^th^ Revision mental health diagnostic codes and groupings.

**eTable 2.** Observed and expected rates, and rate ratios (RR, 95% CI) of outpatient mental health visits in Ontario, March 2020 to February 2021, by material deprivation quintiles.

**eTable 3.** Observed and expected rates, and rate ratios (RR, 95% CI) of outpatient mental health visits in Ontario, March 2020 to February 2021, by immigrant status.

**eTable 4.** Observed and expected rates, and rate ratios (RR, 95% CI) of outpatient mental health visits in Ontario, March 2020 to February 2021, by rurality.

**eTable 1.** Ontario Health Insurance Plan and International Classification of Diseases, 8^th^ Revision mental health diagnostic codes and groupings.

| **Outpatient visit diagnostic codes, Ontario Health Insurance Plan** | | |
| --- | --- | --- |
| **Category** | **Diagnostic Code** | **Description** |
| **Psychotic Disorders** | 295 | Schizophrenia |
|  | 296 | Manic-depressive psychoses, involutional melancholia |
|  | 297 | Other paranoid states |
|  | 298 | Other psychoses |
| **Mood and anxiety Disorders** | 300 | Anxiety neurosis, hysteria, neurasthenia, obsessive-compulsive neurosis, reactive depression |
|  | 301 | Personality disorders |
|  | 302 | Sexual deviations |
|  | 306 | Psychosomatic illness |
|  | 309 | Adjustment reaction |
|  | 311 | Depressive disorder |
| **Substance Use Disorders** | 303 | Alcoholism |
|  | 304 | Drug dependence |
| **Social Problems** | 897 | Economic problems |
|  | 898 | Marital difficulties |
|  | 899 | Parent-child problems |
|  | 900 | Problems with aged parents or in-laws |
|  | 901 | Family disruption/divorce |
|  | 902 | Education problems |
|  | 904 | Social maladjustment |
|  | 905 | Occupational problems |
|  | 906 | Legal problems |
|  | 909 | Other problems of social adjustment |
| **Neuro-developmental and other concerns** | 291 | Alcoholic psychosis, delirium tremens, Korsakov's psychosis |
|  | 292 | Drug psychosis |
|  | 299 | Childhood psychoses (e.g., autism) |
|  | 307 | Habit spasms, tics, stuttering, tension headaches, anorexia nervosa, sleep disorders, |
|  | 313 | Behaviour disorders of childhood and adolescence |
|  | 314 | Hyperkinetic syndrome of childhood |
|  | 315 | Specified delays in development (e.g., dyslexia, dyslalia, motor retardation) |
| **Other mental health fee codes without diagnostic grouping** | **Fee code** | **Description** |
|  | K122 | Developmental and/or behavioural care - individual developmental and/or behavioural care |
|  | K123 | Developmental and/or behavioural care - family developmental and/or behavioural care |
|  | K704 | Paediatric outpatient case conference |
| **Virtual Codes** | Virtual visits were defined as those with either a location code indicating a virtual visit or that included any of the following virtual supplemental codes that were accompanied by an above mental health or addiction (MHA) diagnosis code (with the exception of K082 that does not require a MHA diagnosis code to be considered an MHA visit: B099, B100, B200, B101, B201, B102, B202, B103, B203, B209, K080, K081, K083 | |

| **eTable 2.**  **Observed and expected rates, and rate ratios (RR, 95% CI) of outpatient mental health visits in Ontario, March 2020 to February 2021 by material deprivation quintiles.** All rates are monthly visit rates per 1000 population. | | | | | | | | | | | | | |  |  |
| --- | --- | --- | --- | --- | --- | --- | --- | --- | --- | --- | --- | --- | --- | --- | --- |
|  | **Material Deprivation**  **Q1 (least deprived)** | | | **Q2** | | | **Q3** | | | **Q4** | | | **Material Deprivation**  **Q5 (most deprived)** | | |
| **Month** | **Observed rate** | **Expected rate** | **Rate Ratio**  **(95% CI)** | **Observed rate** | **Expected**  **rate** | **Rate Ratio**  **(95% CI)** | **Observed rate** | **Expected**  **rate** | **Rate Ratio**  **(95% CI)** | **Observed rate** | **Expected rate** | **Rate Ratio**  **(95% CI)** | **Observed rate** | **Expected rate** | **Rate Ratio**  **(95% CI)** |
| Overall | 7.5 | 7.2 | 1.04 (1.02, 1.06) | 6.9 | 6.7 | 1.03 (1.01, 1.05) | 6.6 | 6.6 | 1.00 (0.96, 1.03) | 6.6 | 6.9 | 0.96 (0.94, 0.98) | 6.8 | 7.1 | 0.95 (0.92, 0.98) |
| March 2020 | 6.4 | 7.3 | 0.88 (0.87, 0.90) | 6.0 | 6.9 | 0.82 (0.81, 0.83) | 5.9 | 6.8 | 0.86 (0.83, 0.89) | 5.9 | 7.1 | 0.83 (0.82, 0.84) | 6.3 | 7.4 | 0.85 (0.82, 0.88) |
| April 2020 | 6.4 | 7.5 | 0.85 (0.83, 0.86) | 5.7 | 7.0 | 0.88 (0.87, 0.89) | 5.7 | 7.0 | 0.81 (0.79, 0.84) | 5.6 | 7.2 | 0.78 (0.78, 0.79) | 5.8 | 7.6 | 0.76 (0.74, 0.79) |
| May 2020 | 6.7 | 7.4 | 0.91 (0.89, 0.92) | 6.1 | 6.9 | 0.91 (0.90, 0.92) | 5.8 | 6.8 | 0.86 (0.82, 0.89) | 5.9 | 7.1 | 0.83 (0.81, 0.85) | 6.1 | 7.4 | 0.82 (0.78, 0.85) |
| June 2020 | 6.5 | 7.0 | 0.93 (0.92, 0.94) | 6.0 | 6.6 | 1.11 (1.10, 1.13) | 5.7 | 6.4 | 0.88 (0.87, 0.90) | 5.7 | 6.6 | 0.86 (0.85, 0.87) | 5.9 | 6.9 | 0.84 (0.82, 0.87) |
| July 2020 | 6.5 | 5.8 | 1.12 (1.09, 1.15) | 6.0 | 5.4 | 1.11 (1.07, 1.15) | 5.8 | 5.5 | 1.06 (1.03, 1.09) | 5.8 | 5.7 | 1.02 (0.99, 1.04) | 6.0 | 6.0 | 1.00 (0.97, 1.03) |
| August 2020 | 6.8 | 6.1 | 1.12 (1.09, 1.15) | 6.3 | 5.7 | 1.13 (1.10, 1.15) | 5.9 | 5.6 | 1.05 (1.01, 1.09) | 6.0 | 5.8 | 1.04 (0.99, 1.08) | 6.0 | 6.0 | 0.99 (0.96, 1.02) |
| September 2020 | 7.7 | 6.8 | 1.14 (1.11, 1.18) | 7.1 | 6.3 | 1.15 (1.13, 1.17) | 6.8 | 6.2 | 1.10 (1.07, 1.14) | 6.8 | 6.6 | 1.04 (1.02, 1.06) | 7.0 | 6.9 | 1.01 (0.98, 1.03) |
| October 2020 | 8.7 | 7.6 | 1.15 (1.13, 1.17) | 8.1 | 7.0 | 1.16 (1.13, 1.18) | 7.6 | 7.0 | 1.10 (1.08, 1.11) | 7.7 | 7.3 | 1.05 (1.04, 1.06) | 7.8 | 7.6 | 1.03 (1.00, 1.06) |
| November 2020 | 9.7 | 8.2 | 1.18 (1.16, 1.21) | 8.8 | 7.6 | 1.12 (1.10, 1.13) | 8.4 | 7.5 | 1.13 (1.11, 1.15) | 8.5 | 7.8 | 1.09 (1.08, 1.10) | 8.6 | 8.0 | 1.07 (1.04, 1.10) |
| December 2020 | 6.9 | 6.1 | 1.12 (1.10, 1.14) | 6.3 | 5.7 | 1.14 (1.12, 1.16) | 6.0 | 5.5 | 1.08 (1.05, 1.12) | 5.8 | 5.7 | 1.01 (0.98, 1.04) | 6.0 | 5.9 | 1.02 (0.98, 1.06) |
| January 2021 | 8.9 | 7.8 | 1.14 (1.13, 1.16) | 8.4 | 7.4 | 1.14 (1.13, 1.16) | 8.0 | 7.3 | 1.09 (1.05, 1.14) | 7.8 | 7.5 | 1.05 (1.03, 1.06) | 8.0 | 7.8 | 1.03 (0.99, 1.08) |
| February 2021 | 8.6 | 7.5 | 1.14 (1.13, 1.15) | 8.1 | 7.1 | 0.82 (0.81, 0.83) | 7.7 | 7.0 | 1.10 (1.06, 1.13) | 7.8 | 7.2 | 1.08 (1.06, 1.09) | 7.8 | 7.6 | 1.02 (0.98, 1.07) |
|  |  |  |  |  |  |  |  |  |  |  |  |  |  |  |  |

CI, confidence interval; Q, quintile; RR, rate ratio.

| **eTable 3.** **Observed and expected rates, and rate ratios (RR, 95% CI) of outpatient mental health visits in Ontario, March 2020 to February 2021, by immigrant status.** All rates are monthly visit rates per 1000 population. | | | | | | | | | | | | |
| --- | --- | --- | --- | --- | --- | --- | --- | --- | --- | --- | --- | --- |
|  | | | | | | | | | | | | |
|  | **Immigrants** | | |  | **Refugees** | | |  | **Non-immigrants** | | |  |
|  | **Observed rate** | **Expected**  **rate** | **Rate Ratio**  **(95% CI)** |  | **Observed rate** | **Expected rate** | **Rate Ratio**  **(95% CI)** |  | **Observed rate** | **Expected rate** | **Rate Ratio**  **(95% CI)** |  |
| Overall | 4.3 | 4.0 | 1.07 (1.02, 1.13) |  | 3.6 | 3.7 | 0.98 (0.89, 1.09) |  | 7.2 | 7.1 | 1.00 (0.98, 1.02) |  |
| March 2020 | 3.8 | 4.3 | 0.88 (0.85, 0.90) |  | 3.3 | 4.0 | 0.82 (0.75, 0.89) |  | 6.4 | 7.4 | 0.87 (0.85, 0.88) |  |
| April 2020 | 3.4 | 4.3 | 0.80 (0.76, 0.83) |  | 3.0 | 3.9 | 0.77 (0.70, 0.86) |  | 6.1 | 7.5 | 0.82 (0.80, 0.83) |  |
| May 2020 | 3.7 | 4.2 | 0.89 (0.86, 0.91) |  | 3.0 | 3.8 | 0.77 (0.67, 0.88) |  | 6.4 | 7.4 | 0.87 (0.85, 0.89) |  |
| June 2020 | 3.9 | 3.9 | 1.01 (0.98, 1.04) |  | 3.0 | 3.6 | 0.84 (0.78, 0.90) |  | 6.2 | 7.0 | 0.89 (0.88, 0.90) |  |
| July 2020 | 3.8 | 3.3 | 1.14 (1.07, 1.21) |  | 3.5 | 3.2 | 1.10 (1.02, 1.19) |  | 6.3 | 5.9 | 1.07 (1.04, 1.09) |  |
| August 2020 | 4.1 | 3.2 | 1.26 (1.25, 1.27) |  | 3.5 | 3.0 | 1.16 (1.06, 1.26) |  | 6.4 | 6.1 | 1.06 (1.03, 1.10) |  |
| September 2020 | 4.4 | 3.6 | 1.22 (1.17, 1.27) |  | 3.5 | 3.4 | 1.04 (0.97, 1.12) |  | 7.4 | 6.8 | 1.09 (1.07, 1.11) |  |
| October 2020 | 4.8 | 4.0 | 1.20 (1.12, 1.28) |  | 4.0 | 3.8 | 1.06 (0.95, 1.19) |  | 8.3 | 7.5 | 1.10 (1.09, 1.11) |  |
| November 2020 | 5.1 | 4.3 | 1.17 (1.09, 1.26) |  | 4.2 | 4.1 | 1.04 (0.90, 1.18) |  | 9.2 | 8.1 | 1.14 (1.12, 1.15) |  |
| December 2020 | 3.9 | 3.4 | 1.16 (1.07, 1.25) |  | 3.4 | 3.2 | 1.07 (0.96, 1.19) |  | 6.4 | 6.0 | 1.08 (1.05, 1.10) |  |
| January 2021 | 5.6 | 4.5 | 1.24 (1.16, 1.33) |  | 4.8 | 4.1 | 1.18 (1.04, 1.34) |  | 8.6 | 7.9 | 1.09 (1.07, 1.11) |  |
| February 2021 | 5.2 | 4.4 | 1.18 (1.13, 1.23) |  | 4.0 | 3.9 | 1.03 (0.92, 1.15) |  | 8.3 | 7.6 | 1.10 (1.08, 1.12) |  |

| **eTable 4.** **Observed and expected rates, and rate ratios (RR, 95% CI) of outpatient mental health visits in Ontario, March 2020 to February 2021, by rurality.** All rates are monthly visit rates per 1000 population. | | | | | | | | | |
| --- | --- | --- | --- | --- | --- | --- | --- | --- | --- |
|  | | | | | | | | | |
|  | **Rural** | | |  | **Urban** | | |  |  |
|  | **Observed rate** | **Expected**  **rate** | **Rate Ratio**  **(95% CI)** |  | **Observed rate** | **Expected rate** | **Rate Ratio**  **(95% CI)** |  |  |
| Overall | 5.8 | 5.9 | 0.99 (0.97, 1.01) |  | 7.0 | 7.0 | 1.00 (0.98, 1.02) |  |  |
| March 2020 | 5.4 | 6.1 | 0.88 (0.87, 0.89) |  | 6.2 | 7.2 | 0.86 (0.85, 0.87) |  |  |
| April 2020 | 5.1 | 6.2 | 0.82 (0.82, 0.83) |  | 5.9 | 7.3 | 0.81 (0.79, 0.82) |  |  |
| May 2020 | 5.1 | 6.2 | 0.83 (0.81, 0.84) |  | 6.2 | 7.2 | 0.87 (0.84, 0.89) |  |  |
| June 2020 | 4.9 | 5.7 | 0.86 (0.85, 0.87) |  | 6.1 | 6.8 | 0.89 (0.88, 0.91) |  |  |
| July 2020 | 4.8 | 4.8 | 1.00 (0.99, 1.01) |  | 6.2 | 5.8 | 1.07 (1.05, 1.10) |  |  |
| August 2020 | 5.1 | 5.0 | 1.03 (1.02, 1.04) |  | 6.3 | 5.9 | 1.07 (1.04, 1.10) |  |  |
| September 2020 | 6.2 | 5.7 | 1.07 (1.05, 1.10) |  | 7.2 | 6.6 | 1.09 (1.07, 1.11) |  |  |
| October 2020 | 6.8 | 6.2 | 1.10 (1.09, 1.11) |  | 8.1 | 7.4 | 1.10 (1.09, 1.11) |  |  |
| November 2020 | 7.4 | 6.6 | 1.12 (1.12, 1.13) |  | 9.0 | 7.9 | 1.13 (1.12, 1.15) |  |  |
| December 2020 | 5.3 | 4.9 | 1.08 (1.03, 1.13) |  | 6.3 | 5.9 | 1.07 (1.05, 1.10) |  |  |
| January 2021 | 7.0 | 6.5 | 1.07 (1.04, 1.10) |  | 8.4 | 7.7 | 1.09 (1.07, 1.12) |  |  |
| February 2021 | 6.9 | 6.2 | 1.11 (1.10, 1.12) |  | 8.1 | 7.4 | 1.10 (1.08, 1.12) |  |  |
